# Large and Tunable Polar-Toroidal Coupling in Ferroelectric Composite Nanowires toward Superior Electromechanical Responses

**DOI:** 10.1038/srep11165

**Published:** 2015-06-23

**Authors:** W. J. Chen, Yue Zheng, Biao Wang

**Affiliations:** 1 State Key Laboratory of Optoelectronic Materials and Technologies, School of Physics and Engineering, Sun Yat-sen University, Guangzhou 510275, China; 2 Department of Mechanical Engineering, Northwestern University, Evanston, Illinois 60208, USA; 3 Micro & Nano Physics and Mechanics Research Laboratory, School of Physics and Engineering, Sun Yat-sen University, Guangzhou 510275, China

## Abstract

The collective dipole behaviors in (BaTiO_3_)_*m*_/(SrTiO_3_)_*n*_ composite nanowires are investigated based on the first-principles-derived simulations. It demonstrates that such nanowire systems exhibit intriguing dipole orders, due to the combining effect of the anisotropic electrostatic interaction of the nanowire, the SrTiO_3_-layer-modified electrostatic interaction and the multiphase ground state of BaTiO_3_ layer. Particularly, a strong polar-toroidal coupling that is tunable by the SrTiO_3_-layer thickness, temperature, external strains and electric fields is found to exist in the nanowires, with the appearance of fruitful dipole states (including those being purely polar, purely toroidal, both polar and toroidal, or distorted toroidal) and phase boundaries. As a consequence, an efficient cross control of the toroidal (polar) order by static (curled) electric field, and superior piezoelectric and piezotoroidal responses, can be achieved in the nanowires. The result provides new insights into the collective dipole behaviors in nanowire systems.

Ordering of the spin, charge, lattice or other degrees of freedom are fundamental phenomena in condensed matters, and endow materials with an incredible variety of properties. For example, the cooperative alignment of electric dipoles (spins) leads to ferroelectricity (ferromagnetism), which forms the basis of many device designs, such as memories, sensors and actuators. The coexistence of different orders in a material or structure, namely a multi-order state, is promising in developing multi-state devices. An even more fascinating point of a multi-order state lies in the coupling of different orders^1^,^2^, leading to cross control of the properties, e.g., using electric field to control magnetic properties or reversely using magnetic field to control ferroelectricity based on the magnetoelectric effect in multiferroic materials and structures^3^,^4^,^5^.

Recently, interests have been attracted by complex dipolar states with toroidal order, particularly those found in zero-dimensional magnets^6^,^7^ and ferroelectrics^8^,^9^. Being a special type of dipolar ordering, the natural coupling of toroidal dipolar states with curled fields brings them distinct features from conventional polar states^10^,^11^,^12^,^13^,^14^,^15^. Inspired by the terms “multi-order” and “multiferroic”, it is legitimate to pay attention to multi-order states with coexisting polar and toroidal orders (i.e., with both nonzero polarization and toroidization). Dipolar systems with such polar-toroidal multi-order states (PTMO states) should also be promising in multi-state devices and may carry large coupling behaviors between the polar and toroidal orders, leading to an efficient cross control of toroidal (polar) order by static (curled) field^16^,^17^,^18^,^19^. A discovery of dipolar systems with large polar-toroidal coupling and an understanding their features under external fields is thus of both academic interests and practical significance.

PTMO states are likely to be found in two types of ferroelectric nanosystems. The first type nanosystems are those composite structures consisting of polar components and toroidal components, such as nanodots embedded in polar matrix^20^ or epitaxial nanofilm-dot system^19^. However, the PTMO states reported in these systems seems only exist in a very narrow range of conditions. Alternatively, PTMO state may be achieved in nanosystems under suitable screening condition, for the important role of screening condition in the appearance of polar/toroidal orders in ferroelectrics. It is a natural strategy to look for PTMO state in systems under a screening condition among open-circuit condition and short-circuit condition, where the system is near the critical point of toroidal order and polar order. However, previous studies showed that many ferroelectric nanosystems keep adopting a single-order state (i.e., either polar or toroidal) when the screening factor changes^12^,^21^,^22^. Such an absence of PTMO state is actually due to the fact that the studied ferroelectrics are with a bulk tetragonal ground state and/or under an isotopic screening condition. Beyond this limitation, e.g., for systems made of materials with a bulk multiphase ground state (e.g., BaTiO_3_ and KbNO_3_) and under an anisotropic screening condition, PTMO states may be developed. Indeed, via a first-principles-based simulation, recently Louis *et al.* have found a PTMO state in nanocomposites made of periodic arrays of BaTiO_3_ nanowires embedded in a SrTiO_3_ matrix^17^. Interestingly, such systems were further shown to present an optical activity which can be well controlled by external electric field^18^. More recently, a phase-field simulation also showed that nanodot of multiphase BaTiO_3_ exhibit a PTMO state under axial screening condition^23^. We would like to point out that, among these ferroelectric nanosystems, ferroelectric nanowires in materials with a bulk multiphase ground state are the promising ones to show PTMO states and large polar-toroidal coupling, as it is intrinsically associated with an anisotropic screening condition. Nevertheless, despite the findings of the above mentioned works, very little is known on the controllability of polar-toroidal coupling in ferroelectric nanosystems, not to mention its impact on other properties such as the electromechanical responses of systems.

Using first-principles-derived simulations, here we show that (BaTiO_3_)_*m*_/(SrTiO_3_)_*n*_ nanowires (Fig. 1a) exhibit a large and tunable polar-toroidal coupling, which can lead to fruitful dipole states (including those being purely polar, purely toroidal, both polar and toroidal, or distorted toroidal) and phase boundaries, due to a combining effect of the anisotropic electrostatic interaction of the nanowire, the SrTiO_3_-layer-modified electrostatic interaction and the multiphase ground state of BaTiO_3_ layer (Fig. 1b). In the formula (BaTiO_3_)_*m*_/(SrTiO_3_)_*n*_, *m* and *n* refer to the thickness, in unit cells, of the (001) BaTiO_3_ and (001) SrTiO_3_ layers, respectively. In the nanowires, the polar and toroidal orders are intimately coupled as they are both carried by the same degree of freedom (i.e., electric dipole), similar with the ferroelectric-ferroelastic state in ferroelectrics where the polar and strain orders are both dipole related. As further revealed by the features of electrical and mechanical responses of the nanowires under external fields, such polar-toroidal coupling brings us an efficient cross control of the toroidal (polar) order by static (curled) electric field, as well as superior piezoelectric and piezotoroidal responses in the nanowires.

## Results

### Dipole states of nanowires during a cooling-down process

**Figure 2:**
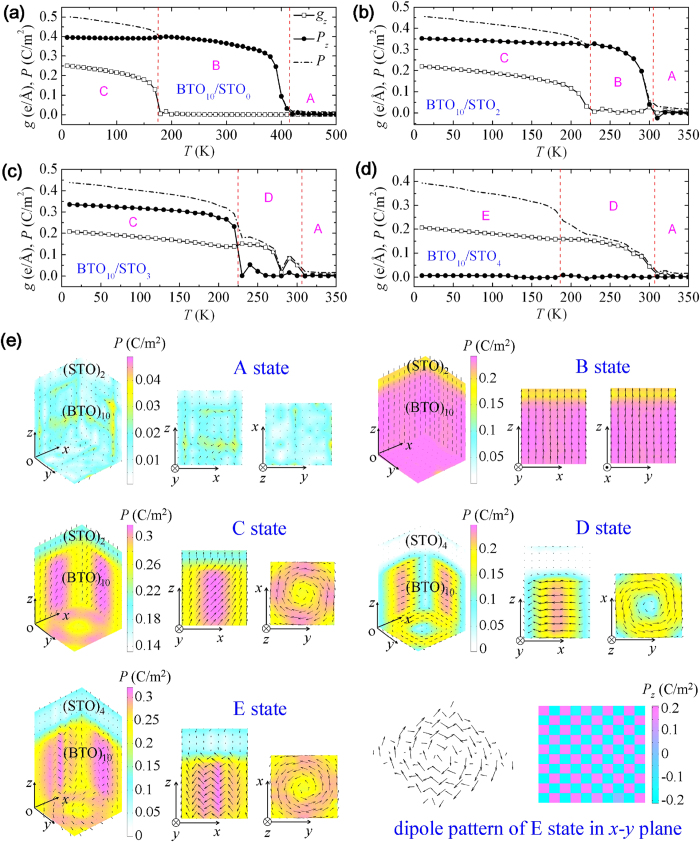
Dipole states of nanowires during a cooling-down process. Evolution of toroidization and polarization in (**a**) (BaTiO_3_)_10_/(SrTiO_3_)_0_, (**b**) (BaTiO_3_)_10_/(SrTiO_3_)_2_, (**c**) (BaTiO_3_)_10_/(SrTiO_3_)_3_ and (**d**) (BaTiO_3_)_10_/(SrTiO_3_)_4_ nanowires. Five dipole states (denoted by *A*, *B*, *C*, *D* and *E*) exist in the nanowire systems. They are the paraelectric state *A*, a purely polar state *B*, a PTMO state *C*, a purely toroidal state *D*, and a distorted toroidal state *E* with the dipoles forming a “polydomain” pattern in the *x*-*y* plane. (**e**) The dipole configurations at selected temperatures of two nanowires, i.e., state *A* of (BaTiO_3_)_10_/(SrTiO_3_)_2_ at 330 K, state *B* of (BaTiO_3_)_10_/(SrTiO_3_)_2_ at 250 K, state *C* of (BaTiO_3_)_10_/(SrTiO_3_)_2_ at 150 K, state *D* of (BaTiO_3_)_10_/(SrTiO_3_)_4_ at 250 K, and state *E* of (BaTiO_3_)_10_/(SrTiO_3_)_4_ at 150 K.

The collective dipole configurations of (BaTiO_3_)_10_/(SrTiO_3_)_*n*_ nanowires with *n* = 0, 2, 3 and 4 and a lateral size being *L*_*x*_ = *L*_*y*_ ≈ 4 nm during a cooling-down process is depicted in Fig. 2. To quantify the dipole state of the nanowires, we calculate both the toroidization **g** and the net polarization **P** as shown in Fig. 2a–d. Up to five dipole states (denoted by *A*, *B*, *C*, *D* and *E*) exist in the nanowire systems. They are (1) the paraelectric state *A* at high enough temperatures in all the nanowires, (2) a purely polar state *B* (*P*_*z*_ ≠ 0 and *g*_*z*_ = 0) in nanowires with *n* ≤ 2 at moderate temperatures, (3) a PTMO state *C* (*P*_*z*_ ≠ 0 and *g*_*z*_ ≠ 0) in nanowires with *n* ≤ 3 at low temperatures, (4) a purely toroidal state *D* (*P*_*z*_ = 0 and *g*_*z*_ ≠ 0) at moderate temperatures in nanowires with *n* ≥3, and (5) a distorted toroidal state *E* with the dipoles forming a “polydomain” pattern in the *x-y* plane (*P*_*z*_ = 0 and *g*_*z*_ ≠ 0) at low temperatures in nanowires with *n* > 3. To be clearer about the domain states, in Fig. 2e we depict the collective dipole configurations at selected temperatures of two nanowires, i.e., state *B* of (BaTiO_3_)_10_/(SrTiO_3_)_2_ at 250 K, state *C* of (BaTiO_3_)_10_/(SrTiO_3_)_2_ at 150K, state *D* of (BaTiO_3_)_10_/(SrTiO_3_)_4_ at 250 K, and state *E* of (BaTiO_3_)_10_/(SrTiO_3_)_4_ at 150 K. It is original and important to show such a variety of dipole states with distinct polar and toroidal ordering exists in composite nanowires.

The appearance of fruitful dipole states results from the combining effect of the anisotropic electrostatic interaction of the nanowire, the SrTiO_3_-layer-modified electrostatic interaction and the multiphase ground state of BaTiO_3_. Specifically, (BaTiO_3_)_10_/(SrTiO_3_)_*n*_ nanowires with a sufficiently thin SrTiO_3_ layer (*n* ≤ 2) at moderate temperatures adopt a *z*-directed polar state *B* that commonly found in many ferroelectric nanowire systems^12^,^22^,^24^ to avoid a large depolarizing energy. Due to the proximity to ferroelectric BaTiO_3_ layers, the SrTiO_3_ layer polarizes by electrostatic effect in a magnitude of polarization smaller than that of the BaTiO_3_ layer, similar to the behavior of (BaTiO_3_)_*m*_/(SrTiO_3_)_*n*_ superlattices^25^,^26^. Interestingly, the nanowires at lower temperatures would not maintain the purely polar state *B* but adopt a PTMO state *C* with a polar order along the *z*-axis and a toroidal order in the *x*-*y* plane. Dipoles of the BaTiO_3_ layer tilt at low temperature due to the rhombohedral ground state of BaTiO_3_. Nevertheless, a state with net in-plane polarization is not allowed by open-circuit the condition. As a result, the tilted dipoles have to rotate around the *z*-axis to form a vortex-like pattern. Such a PTMO state is similar to that found in BaTiO_3_ nanowires embedded in a SrTiO_3_ matrix^17^,^18^ and has also been indicated in BaTiO_3_ nanodots under an anisotropic screening^23^ and in free standing BaTiO_3_ nanowires^27^,^28^. Note also that the dipoles in weak ferroelectric SrTiO_3_ layer also simultaneously exhibit polar and toroidal orders due to the proximity effect, although with smaller polarization and toroidization than the BaTiO_3_ layer.

We notice that the BaTiO_3_/SrTiO_3_ composite structure in previous works^17^,^18^ is 1–3 type, with the one dimensional (1D) BaTiO_3_ nanowires periodically embedded in three dimensional (3D) SrTiO_3_ matrix. The SrTiO_3_ matrix surrounds the side surface of the BaTiO_3_ nanowires and imposes an open-circuit like condition to the nanowires at the side surface. In case of large distance between the nanowires, the dipole state of the individual nanowire is similar to a strained single BaTiO_3_ nanowire. In this work, we focus on an entirely new structure, i.e., a single nanowire system with BaTiO_3_ and SrTiO_3_ layers (or blocks) periodically alternating along the wire direction. The individual BaTiO_3_ and SrTiO_3_ layers are confined in all dimensions, yet interact with others via short-range interfacial interaction and long-range electrostatic interaction along the wire direction. It is an important point of our work to put forward the concept of such a composite nanowire structure to show a large and tunable polar-toroidal coupling.

Moreover, our MC simulations show that nanowires with relatively thick SrTiO_3_ layer (*n* ≥ 3) adopts a purely toroidal state *D* at moderate temperatures rather than the purely polar state *B*, indicating that the SrTiO_3_ layer thickness strongly affects the dipole behavior of the nanowires. This is due to that a thicker SrTiO_3_ layer decreases the layer electrostatic effects that help stabilize polar order and imposes a more open-circuit-like condition to the BaTiO_3_ layer. At lower temperatures, as the dipoles tilt toward *z*-axis, the purely toroidal state is not stable and would change into either a PTMO state *C* (Fig. 2c) or a distorted toroidal state *E* (Fig. 2d). Interestingly, due to the buffering SrTiO_3_ layer, the polydomain in the *x*-*y* plane of state *E* can be so dense that a perfect chessboard-like pattern of the *z* component of dipoles in the *x*-*y* plane appears (Fig. 2e). Further simulation shows that state *E* can be poled into the PTMO state by an applied electric field along the *z*-axis, analogous with the poling of dense 180° domains, indicating that state *E* forms to reduce the depolarizing energy. It is noteworthy that nanowires with thicker SrTiO_3_ layer exhibit similar dipole states with (BaTiO_3_)_10_/(SrTiO_3_)_4_ nanowire (Supplementary Figure S1). A larger thickness of SrTiO_3_ layer lowers the Curie temperature only slightly, as the misfit strain between the SrTiO_3_ and BaTiO_3_ layers increases and acts oppositely with the depolarizing field effect. On the other hand, the transition temperature *E*/*D* states increases with the SrTiO_3_ layer thickness, leading to shrinkage of the stable range of state *D*.

### Cross control of toroidal order by static electric field

**Figure 3:**
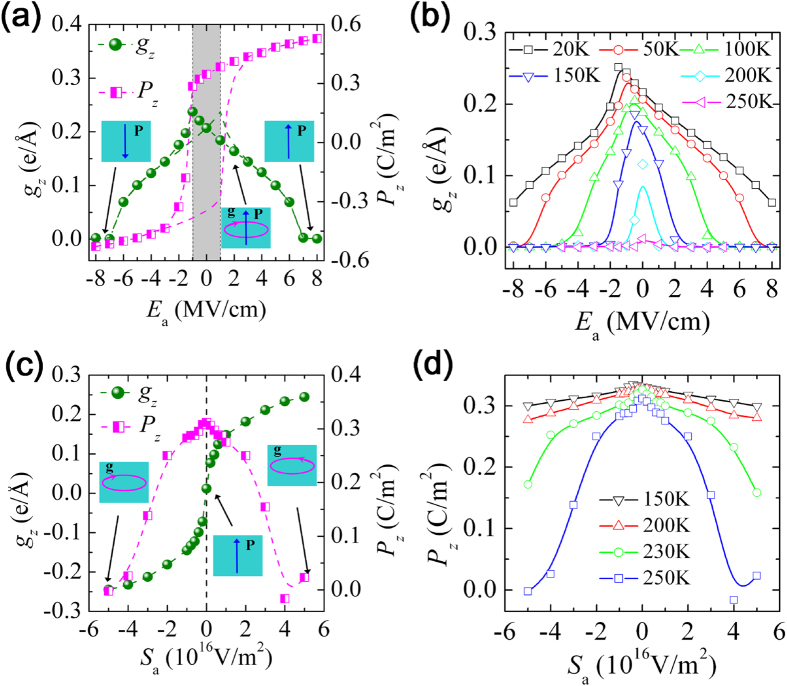
Cross control of toroidal (polar) order by static (curled) electric field. Evolution of dipole states of (BaTiO_3_)_10_/(SrTiO_3_)_2_ nanowire under an external static field **E**_H_ = *E*_a_**e**_***z***_ or a curled field **E**_C_ = *S*_a_**e**_***z***_** **×** r**. The initial dipole states of the nanowire are those obtained during a cooling-down process under zero external fields. (**a**) The toroidization and polarization as functions of *E*_a_ at *T* = 50 K. (**b**) The toroidization as a function of *E*_a_ at different temperatures. (**c**) The toroidization and polarization as functions of *S*_a_ at *T* = 250 K. (**d**) The polarization as a function of *S*_a_ at different temperatures.

The sensitivity of the state stability to SrTiO_3_ layer thickness implies that we may achieve large tunability of the dipole states of the nanowires by applying external fields. The large tunability is evidenced by simulations on the electromechanical responses of the nanowires under external electric and mechanical fields (for more results, see Supplementary Figure S2-S7). Behavior of the (BaTiO_3_)_10_/(SrTiO_3_)_2_ nanowire under an external *z*-directed static electric field **E**_H_ = *E*_a_**e**_*z*_ at *T* = 50 K is depicted in Fig. 3a. Note that, at a given temperature, the nanowire initially adopts a dipole state that was obtained during a cooling-down process under zero external electric field (see Fig. 2b). The nanowire is thus initially in PTMO state *C* at *T* = 50 K. The important observations are following. (1) For such PTMO state with toroidal order, it would transform into a purely polar state under the action of electric field. Different from the complicated path predicted in nanoparticles^29^, this transformation is achieved by the simple collective tilting of dipoles to the *z*-axis. (2) The system’s toroidization exhibits a large and linear response at low field region (see the gray area in Fig. 3a), another remarkable feature of our system that is similar to that of BaTiO_3_ nanowires embedded in a SrTiO_3_ matrix^18^ and in contrast with the small and quadratic response of the nanoparticles^29^. Particularly, for *E*_a_ within the coercive field (~1 MV/cm), the toroidization changes linearly with *E*_a_ (either increases or decreases depending on the direction of the field versus the polarization) in a law of *g*_*z*_(*E*_a_) = *g*_*0*_ − *χE*_a_, where *g*_0_ is the zero-field toroidization and the toroidal susceptibility of static field *χ* is about 2.8 e/V by a linear fitting. Beyond the coercive field, an increase of *E*_a_ monotonously increases the polarization magnitude and decreases the toroidization, with a vanishing of toroidal order at a field of about 7 MV/cm.

Figure 3b depicts dependence of the toroidization of (BaTiO_3_)_10_/(SrTiO_3_)_2_ nanowire on static field *E*_a_ at different temperatures. As expected, the vanishing field of toroidal order significantly decreases as the temperature increases. And more importantly, the susceptibility *χ* increases as the temperature approaches to the phase boundary between polar state *B* and PTMO state *C*. To quantify the susceptibility at small field, we calculate it by the statistical correlation between *g*_*z*_ and *P*_*z*_^12^,^30^, and find that *χ* of the PTMO state *C* monotonously increases with temperature and reaches about 12 e/V at around 210 K (see Supplementary Figure S8a). Similar responses of toroidal order to a static field have been also observed in other nanowire systems. An even larger *χ* is observed in the PTMO state *C* of (BaTiO_3_)_10_/(SrTiO_3_)_3_ nanowire, with a magnitude of over 150 e/V found near the phase boundary between purely toroidal state *D* and PTMO state *C* (Supplementary Figure S8b). These results clearly demonstrate the large and tunable polar-toroidal coupling in our composite nanowire system. It is noteworthy that, in single-order states *B*, *D* and *E*, the calculated statistical correlation between toroidization and polarization is misleading due to the absence of stable polar or toroidal order. A strong coupling of the toroidal order to external static field can still be achieved in toroidal states *D* and *E*, since these states would transform into PTMO state *C* once the field is exerted.

### Cross control of polar order by curled electric field

One may expect, reversely, an efficient control of polar order by curled field in the nanowires. This has been already indicated by the previously calculated toroidal susceptibility at static field as it is also the polar susceptibility at curled field (this can be clearly seen from the Maxwell relation 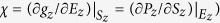. For (BaTiO_3_)_10_/(SrTiO_3_)_2_ nanowire at *T* = 250 K, its evolution of dipole state under a curled electric field **E**_C_ = *S*_a_**e**_*z*_ × **r** is depicted in Fig. 3c. The polarization indeed can be well tuned by the curled field. It shows that the nanowire with a purely polar state *B* (with *P*_*z*_ of about 0.32 C/m^2^) transforms into a purely toroidal state when *S*_a_ exceeds 4 × 10^16^ V/m^2^. Such a large response of net polarization to curled field reflects the easy titling of dipoles. As indicated by the *S*_a_ dependence of the polarization of the (BaTiO_3_)_10_/(SrTiO_3_)_2_ nanowire at different temperatures (Fig. 3d), the nanowire has a largest response to the curled field near the phase boundary of paraelectric state *A* and polar state *B*. For other nanowires with a different thickness of SrTiO_3_ layer, large response to a curled field is also found especially near the temperature region right after the polar order becomes significant. Note that, for nanoparticles under open-circuit condition, the application of curled field can seldom affect their net polarization as it tends to keep a null value^31^; meanwhile, this polar-to-vortex transformation should be common in nanowire systems. However, there is a striking feature resides in our nanowire systems: the polar-to-vortex transformation is much easier to be induced due to modified long-range interaction of dipoles along the *z*-axis induced by the SrTiO_3_ layer and the tetragonal-to-rhombohedral phase transition of bulk BaTiO_3_.

### Piezoelectric and piezotoroidal responses of the nanowires

**Figure 4:**
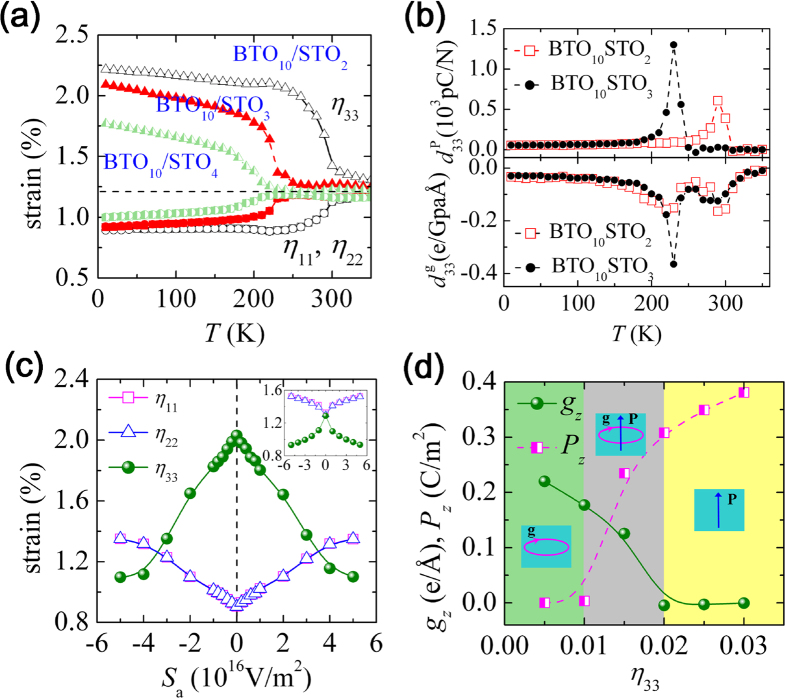
Piezoelectric and piezotoroidal responses of the nanowires. (**a**) The strain state of the (BaTiO_3_)_10_/(SrTiO_3_)_*n*_ nanowires with *n* = 2, 3 and 4 as a function of temperature at zero external fields. (**b**) The calculated piezoelectric 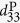 and piezotoroidal 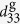 coefficients of (BaTiO_3_)_10_/(SrTiO_3_)_2_ and (BaTiO_3_)_10_/(SrTiO_3_)_3_ nanowires. (**c**) Inverse and (**d**) direct piezotoroidal effect in (BaTiO_3_)_10_/(SrTiO_3_)_2_ nanowire at *T* = 250 K. The nanowire has an initial purely toroidal state. (**c**) The strain state of the nanowire as a function of a curled field **E**_C_ = *S*_a_**e**_***z***_** **×** r**. The insert depicts the strain state of BaTiO_3_ nanodot as a function of *S*_a_ at *T* = 250 K. (**d**) The toroidization and polarization as functions of constrained strain *η*_33_.

We now turn to the electromechanical responses of the nanowires when they are under external electric fields or strain constraint, which are related to the well known piezoelectric and the less reported piezotoroidal effects^12^. Figure 4a depicts the strain state of the (BaTiO_3_)_10_/(SrTiO_3_)_*n*_ nanowires with *n* = 2, 3 and 4 as a function of temperature under zero external fields. For each nanowire, a change of strain state is accompanied with the transition of dipole states. Particularly, a large change of axial strain happens near the phase boundary of paraelectric state and state *B* in the (BaTiO_3_)_10_/(SrTiO_3_)_2_ nanowire, and the phase boundary of state *D* and state *C*(*E*) in (BaTiO_3_)_10_/(SrTiO_3_)_3(4)_ nanowire, indicating large electromechanical responses near these phase boundaries. Figure 4b depicts the calculated piezoelectric 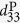 and piezotoroidal 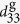 coefficients of the (BaTiO_3_)_10_/(SrTiO_3_)_2_ and (BaTiO_3_)_10_/(SrTiO_3_)_3_ nanowires. Two remarkable observations are as follows. (1) Both nanowires have an overall notable piezoelectric coefficient 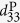 (>50 pC/N) with a peak over 500 pC/N and 1200 pC/N near the phase boundary of polar and nonpolar states. (2) The piezotoroidal coefficient 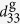 of the nanowires also exhibits a peak near the phase boundary of PTMO state and non-toroidal states, with an overall large value (>0.02 e/GpaǺ even near 0 K). Such an overall large 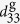 is a result of polar-toroidal coupling, and is an order larger than that previously found in PZT nanodot^12^. Moreover, the peak value of 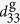 is found comparable with BaTiO_3_ nanodot, which has a large 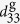 at moderate temperatures due to vortex rotation (see Supplementary Figure S8c).

As previously discussed on the toroidal susceptibility, the calculated piezotoroidal coefficient of (BaTiO_3_)_10_/(SrTiO_3_)_2_ nanowire in polar state *A* (Fig. 4b) is misleading as the state is absent of toroidal order. A large inverse piezotoroidal effect of the nanowire in polar state *A* is actually hinted by the previous results on the large control of net polarization by a curled field (Fig. 3c). To verify this, in Fig. 4c, we calculate the strain state of the (BaTiO_3_)_10_/(SrTiO_3_)_2_ nanowire as a function of curled field at *T* = 250 K, from which a large inverse piezotoroidal effect is indeed seen. The nanowire exhibits a sharp response peak at small field, similar with the strain response at small field of the BaTiO_3_ nanodot undergoing vortex rotation (see the insert). Importantly, the strain response to the curled field of the nanowire exhibits a much more linear feature than that of the nanodot. As the field reaches 5 × 10^16^ V/m^2^, a strain change of about 0.95% is achieved in the nanowire, which is much larger than the 0.45% strain change of the nanodot. Strong direct piezotoroidal responses are also found in the nanowires. As a typical result, for the (BaTiO_3_)_10_/(SrTiO_3_)_2_ nanowire at *T* = 250 K (Fig. 4d), a constraint on strain *η*_33_ largely affects the nanowire’s toroidization and polarization, leading to transformations between PTMO state, purely toroidal state and purely polar state.

## Discussion

First-principles-derived simulations are preformed to investigate the dipole state in (BaTiO_3_)_*m*_/(SrTiO_3_)_*n*_ composite nanowires. We demonstrate that a large polar-toroidal coupling exists in this entirely new composite system. In this composite nanowire system, the toroidal/polar coupling can be well tuned by the SrTiO_3_-layer thickness, temperature, external strains and electric fields, and brings fruitful dipole states and phase boundaries in the nanowires. Importantly, efficient cross control of the toroidal (polar) order by static (curled) electric field, and superior electromechanical responses can be achieved in the nanowires. We hope that the intriguing dipole behavior of the composite nanowires and their striking features in electrical and electromechanical responses will be experimentally confirmed soon.

Our study sheds several insights into the collective dipole behaviors in ferroelectric nanosystems. Firstly, it emphasizes that nanowire systems are natural candidates for exploring polar-toroidal coupling due to that they are intrinsically associated with an anisotropic screening condition. Polar and toroidal orders can coexist in these systems. This feature is promising in developing multi-state storage, and provides us an efficient cross control of the toroidal (polar) order by static (curled) electric field. Secondly, it demonstrates that fruitful dipole states can be achieved in the composite nanowires by making use of the anisotropic electrostatic interaction of the nanowire, the layer-modified electrostatic interaction and the multiphase ground state of bulk ferroelectric. The layer-modified electrostatic interaction provides an alternative control of the nanowire’s anisotropic screening condition, and indicates fruitful controllability of the dipole behavior in ferroelectric nanosystems under mixed electric boundary conditions^23^. Thirdly, our result reveals some interesting phase boundaries in nanowires, i.e., that between PTMO state and polar state, that between PTMO state and toroidal state, and that between distorted toroidal state and toroidal state. Large electromechanical responses are observed near these boundaries. Different from predicted phase boundary between polar and toroidal states in PZT nanowires^32^, which needs to be induced at a finite field, these phase boundaries naturally exist at zero fields. All these new features of nanowires should be important for the designs of novel nanowire-based devices^33^.

## Methods

We perform the simulations using a first-principles derived effective Hamiltonian of (Ba,Sr)TiO_3_ solid solutions^25^. The energy is written as a function of local modes **u **_*i*_, inhomogeneous strain *η*_ I_, alloy species *σ*_*i*_ (in value of +1 or −1 corresponding to the presence of a Ba or Sr atom), local strain *η*_loc_ resulting from the difference in ionic size between alloy species, and homogenous strain *η*_H_,


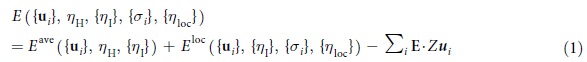


where the first term *E*^ave^ describes the total energy associated with (Ba,Sr)TiO_3_ system in the virtual crystal approximation (VCA)^34^, the second term *E*^loc^ gathers energy associated with alloying effects going beyond the VCA, and the last term takes into account the effect of external electric field **E** (*Z* is the effective charge). Explicit expression of *E*^ave^ and *E*^loc^ can be respectively found in ref. 35,25. All the parameters of the Hamiltonian are extracted from the first-principle calculations on relatively small supercells. Note that, although the Hamiltonian was initially proposed for disordered systems, it has been shown to capture well the ferroelectric properties of (BaTiO_3_)_*m*_/(SrTiO_3_)_*n*_ structures^25^. A depolarizing energy arises due to bounded charges at the side surfaces of nanowire systems. To consider this energy, an efficient dual-space approach based on periodic Green’s function for dipole-dipole interaction in one-dimensional periodic systems is adopted^21^, instead of the Ewald summation method for three-dimensional periodic systems^35^.

Note that the surface polarization might have a significant impact on the collective dipole behavior of the nanowire. To describe this surface effect, an additional energy term has to be added to the Hamiltonian as^36^


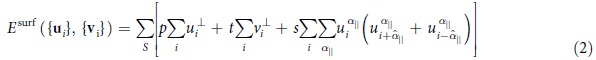


The three terms of Eq. (2) mimic explicit interactions between |the nanowire and the vacuum, with *S* referring to the free surfaces of the nanowire and index *i* running over the B sites closest to the surface. 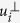 denotes the component of the local mode normal to the surface. *α*_||_ runs over the axes along the surface. 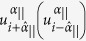 is the *α*_||_ component of the local mode that is the closest from the *i* site along the positive (negative) direction of the *α*_||_ axis. The *p* and *t* parameters quantify how vacuum affects the out-of-plane components of the local modes and inhomogeneous strains near the surface, respectively. *s* characterizes the change, with respect to the bulk, of the short-range interaction between the in-plane components of the local modes near the surface. The *p*, *t*, and *s* parameters can be determined from first-principles calculations on BTO (BaO-terminated) and STO surface (SrO-terminated).

We consider (BaTiO_3_)_*m*_/(SrTiO_3_)_*n*_ nanowires with a sufficiently long length, which is mimicked by means of periodicity along the longitudinal *z* axis. The wires are chosen to be in rectangular shape, with their *x*, *y*, and *z* axes along the pseudocubic [100], [010] and [001] directions of the perovskite structure, respectively. The lengths of simulated supercells along *x* and *y* axes are *L*_*x*_ = *n*_*x*_*a* and *L*_*y*_ = *n*_*y*_*a*, respectively, while the *z*-axis periodic length *L*_*z*_ is *L*_*z*_ = (*m* + *n*)*a*, with *a* ≈ 4 Å being the lattice constant of the primitive five-atom unit cell. We use the energy in Eq. (1) and finite temperature Monte Carlo (MC) simulations to determine the dipole state of the nanowires at specific temperature and external fields (i.e., static or curled electric fields and strain load). An ideal open-circuit condition is imposed to the nanowires. To obtain the ground dipole state, the nanowires are cooled down at zero external fields from a high paraelectric temperature with the temperature decreased by a step of 10 K. External fields are then applied to the nanowires with the initial zero-field dipole state at given temperatures to investigate the electrical and mechanical responses of the nanowires. At each situation, at least 20000 and 60000 MC sweeps are used to equilibrate the system and to get the statistical average, respectively.

We have conducted simulations of nanowires with and without this surface term. Note that due to a lack of values of *p*, *t*, and *s* parameters, we have tried a set of values in the same magnitude with those of PZT surface (AO-terminated) reported in literature^36^. We find that the simulated dipole patterns of the nanowires with and without this surface term are similar, indicating the surface effect has a minor effect on the overall collective dipole behavior in nanowires at this size (see Supplementary Figure S9). This small effect of surface term is consistent with the results of Fu and Bellaiche^8^, who found that the collective dipole behavior of BaTiO_3_ nanodots is not much affected by the surface. We have also conducted simulations on nanowires with different sizes (e.g., nanowires with 12 × 12 unitcells and 14 × 14 in the cross section). The result further indicates that the dipolar orders found in this work are not sensitive to the surface polarization when the size of the nanowire is not too small (see Supplementary Figure S10). This is due to the fact that toroidal order is mainly caused by the depolarization effect and it is a collective behavior. Therefore, the inclusion of a surface term would not alter the main physics of our results. For simplicity, in the mass simulations, we turned off the surface term in Eq. (2).

To characterize the dipole state of the nanowires, both the toroidization 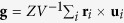 and the net polarization 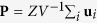 are calculated, where **r**_*i*_ is the displacement vector of the *i*th unitcell and *V* is the supercell volume. Note that, here we have omitted the factor 1/2 in the definition of toroidization **g** and in that of the curled field, i.e., **E**_C_ = **S **× **r**, to ensure that the product **g**·**S** is equivalent to **P**·**E**, as they are all about the interaction between electric field and dipoles. For example, for a system in a curled field *E*_*x*_ = −*S*_*z*_*y* and *E*_*y*_ = *S*_*z*_*x*, the interaction energy is given by
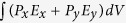
and should be equal to *VS*_*z*_* g*_*z*_.

The susceptibility at small field is calculated according to the statistical correlation between *g*_*z*_ and *P*_*z*_^12^,^25^. Specifically, we use the relationship *χ* = *Vβ* <Δ*P*_*z*_Δ*g*_*z*_>, where Δ*Y* = *Y* *− *〈*Y*〉, 〈*Y*〉 the thermodynamic average of *Y*, and *β* = 1/*kT* with *k* being the Boltzmann constant. For a system under a static field along the *z* axis and a curled field in the *xy* plane, its thermodynamic potential is given by *G* = *U *− *P*_*z*_*E*_*z*_ − *S*_*z*_ *g*_*z*_. The expression of *χ* can be obtained by differentiation of the equation 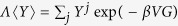, where **Λ** is the partition function. The piezoelectric and the piezotoroidal coefficients 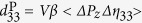 and 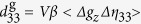 can be deduced in a similar way.

## Additional Information

**How to cite this article**: Chen, W. J. *et al.* Large and Tunable Polar-Toroidal Coupling in Ferroelectric Composite Nanowires toward Superior Electromechanical Responses. *Sci. Rep.* **5**, 11165; doi: 10.1038/srep11165 (2015).

## Supplementary Material

Supplementary Information

## Figures and Tables

**Figure 1:**
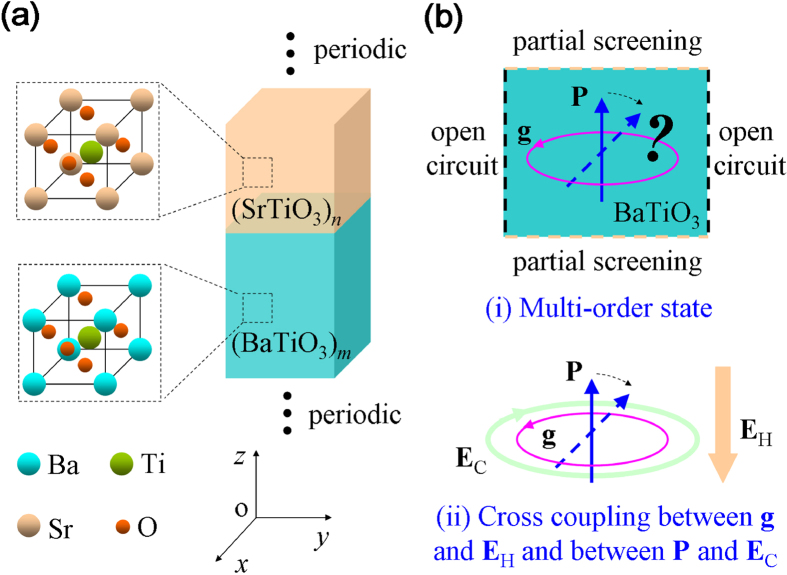
Schematics of the model system and the basic idea of this investigation. (**a**) A periodic (BaTiO_3_)_*m*_/(SrTiO_3_)_*n*_ composite nanowire. (**b**) In the nanowire, the BaTiO_3_ region is under partial screening along one direction and open-circuit condition along the other directions. (i) A multi-order state with coexistence of polar and toroidal orders and (ii) cross coupling between toroidization **g** and static electric field **E**_H_, and between polarization **P** and curled electric field **E**_C_ may be achieved.
