# Supplementary material for: Large and Tunable Polar-Toroidal Coupling in Ferroelectric Composite Nanowires toward Superior Electromechanical Responses
Source: Sci Rep. 2015 Jun 23;5:11165. doi: 10.1038/srep11165 (PMC4477413; doi:10.1038/srep11165)

Supplementary Information for

“Large and Tunable Polar-Toroidal Coupling in Ferroelectric Composite Nanowires toward Superior Electromechanical Responses”

Weijin Chen,[1,3] Yue Zheng[1,2,3] and Biao Wang[1]

[1]*State Key Laboratory of Optoelectronic Materials and Technologies, School of Physics and Engineering, Sun Yat-sen University, Guangzhou 510275, China*

[2]*Department of Mechanical Engineering, Northwestern University, Evanston, Illinois 60208, USA.*

[3]*Micro & Nano Physics and Mechanics Research Laboratory, School of Physics and Engineering, Sun Yat-sen University, Guangzhou 510275, China*

Figure S1| Evolution of toroidization and polarization during a cooling-down process in (a) a 10×10×10 $BaTiO_3$ nanodot, (b) $(BaTiO_3)_{10}/(SrTiO_3)_6$, and (c) $(BaTiO_3)_{10}/(SrTiO_3)_8$ nanowires. "*A*" denotes the paraelectric state, "*D*" a purely toroidal state, "*E*" a distorted toroidal state, and "*F*" the rhombohedral vortex state. (d) Dipole configuration of the rhombohedral vortex state *F*.

Figure S2| Evolution of dipole states of $(BaTiO_3)_{10}/(SrTiO_3)_2$ nanowire under external electric fields or strain constraint. (a) The initial dipole states of the nanowire obtained during a cooling-down process under zero external fields. (b) Under an external static field $\mathbf{E}_H=E_a\mathbf{e_z}$ with E_a=1MV/cm. (c) Under a curled field $\mathbf{E}_C=S_a\mathbf{e_z}\times\mathbf{r}$ with $S_a=5\times10^{16}$V/m^2. (d) Under a constraint of axial strain $\eta_{33}=0.01$. "*A*" denotes the paraelectric state, "*B*" a purely polar state, "*C*" a PTMO state, and "*D*" a purely toroidal state.

Figure S3| Evolution of dipole states of $(BaTiO_3)_{10}/(SrTiO_3)_3$ nanowire under external electric fields or strain constraint. (a) The initial dipole states of the nanowire obtained during a cooling-down process under zero external fields. (b) Under an external static field $\mathbf{E}_H=E_a\mathbf{e_z}$ with E_a=1MV/cm. (c) Under a curled field $\mathbf{E}_C=S_a\mathbf{e_z}\times\mathbf{r}$ with $S_a=5\times10^{16}$V/m^2. (d) Under a constraint of axial strain $\eta_{33}=0.01$. "*A*" denotes the paraelectric state, "*B*" a purely polar state, "*C*" a PTMO state, and "*D*" a purely toroidal state.

Figure S4| Evolution of toroidization component g_z and polarization component P_z of $(BaTiO_3)_{10}/(SrTiO_3)_2$ nanowire under external fields, i.e., an external static field $\mathbf{E}_H=E_a\mathbf{e_z}$, a curled field $\mathbf{E}_C=S_a\mathbf{e_z}\times\mathbf{r}$ or a constraint of axial strain η_{33}. The initial dipole states of the nanowire are those obtained during a cooling-down process under zero external fields. (a) The toroidization and (b) polarization as a function of temperature at different E_a. (c) The toroidization and (d) polarization as a function of temperature at different S_a. (e) The toroidization and (f) polarization as a function

of temperature at different constraint of axial strain η_{33} .

Figure S5| Evolution of toroidization component g_z and polarization component P_z of $(BaTiO_3)_{10}/(SrTiO_3)_3$ nanowire under external fields, i.e., an external static field $\mathbf{E}_H=E_a\mathbf{e_z}$, a curled field $\mathbf{E}_C=S_a\mathbf{e_z}\times\mathbf{r}$ or a constraint of axial strain η_{33}. The initial dipole states of the nanowire are those obtained during a cooling-down process under zero external fields. (a) The toroidization and (b) polarization as a function of temperature at different E_a. (c) The toroidization and (d) polarization as a function of temperature at different S_a. (e) The toroidization and (f) polarization as a function of temperature at different constraint of axial strain η_{33} .

Figure S6| Dipole configuration evolution of dipole states of $(BaTiO_3)_{10}/(SrTiO_3)_2$ nanowire under various external fields. (a) Purely polar state *B* under a curled field at *T*=250K. The increasing field finally transforms the initial state into a purely toroidal state *D*. (b) Purely polar state *B* under a relatively compressive axial strain constraint at *T*=250K. The increasing strain constraint finally transforms the initial state into a purely toroidal state *D*. (c) PTMO state *C* under a static field at *T*=150K. The increasing field finally transforms the initial state into a purely polar state *B*. (d) PTMO state *C* of under a relatively tensile strain constraint at *T*=200K. The increasing strain constraint finally transforms the initial state into a purely polar state *B*. (e) PTMO state *C* under a relatively compressive strain constraint at *T*=200K. The increasing strain constraint finally transforms the initial state into a purely toroidal state *D*.

Figure S7| Dipole configuration evolution of dipole states of $(BaTiO_3)_{10}/(SrTiO_3)_3$ and $(BaTiO_3)_{10}/(SrTiO_3)_4$ nanowires under various external fields. (a) Purely toroidal state *D* of $(BaTiO_3)_{10}/(SrTiO_3)_3$ nanowire under a static field at *T*=250K. The increasing field finally transforms the initial state into a purely polar state *B*. (b) Purely toroidal state *D* of $(BaTiO_3)_{10}/(SrTiO_3)_3$ nanowire under a relatively tensile

axial strain constraint at T=250K. The increasing tensile strain constraint finally transforms the initial state into a purely polar state B. (c) Distorted toroidal state E of $(BaTiO_3)_{10}/(SrTiO_3)_4$ nanowire under a static field at T=150K. The increasing field finally transforms the initial state into a purely polar state B. (d) Distorted toroidal state E of $(BaTiO_3)_{10}/(SrTiO_3)_4$ nanowire under a relatively compressive axial strain constraint at T=150K. The increasing strain constraint finally transforms the initial state into a purely polar state B. (e) Distorted toroidal state E of $(BaTiO_3)_{10}/(SrTiO_3)_4$ nanowire under a relatively tensile axial strain constraint at T=150K. The increasing strain constraint finally transforms the initial state into a dense 180° dipole state without toroidal order in the x-y plane.

Figure S8| Calculated toroidal susceptibility χ in PTMO state C of (a) $(BaTiO_3)_{10}/(SrTiO_3)_2$ and (b) $(BaTiO_3)_{10}/(SrTiO_3)_3$ nanowires. (c) Piezotoroidal d_{33}^{g} coefficient of a 10×10×10 $BaTiO_3$ nanodot.

Figure S9| The dipole patterns of $(BaTiO_3)_{10}/(SrTiO_3)_4$ nanowires at T=250K (a) without and (b), (c) with considering the surface energy term Eq. (2). (b) p=0.015, t=0.007, s=-0.004 and (c) p=-0.015, t=0.007, s=-0.004 (in atomic units).

Figure S10| The dipole patterns of $(BaTiO_3)_{10}/(SrTiO_3)_4$ nanowires with different size of cross section at T=250K. (a) 12×12 unitcells and (b) 14×14 unitcells.

Figure S1

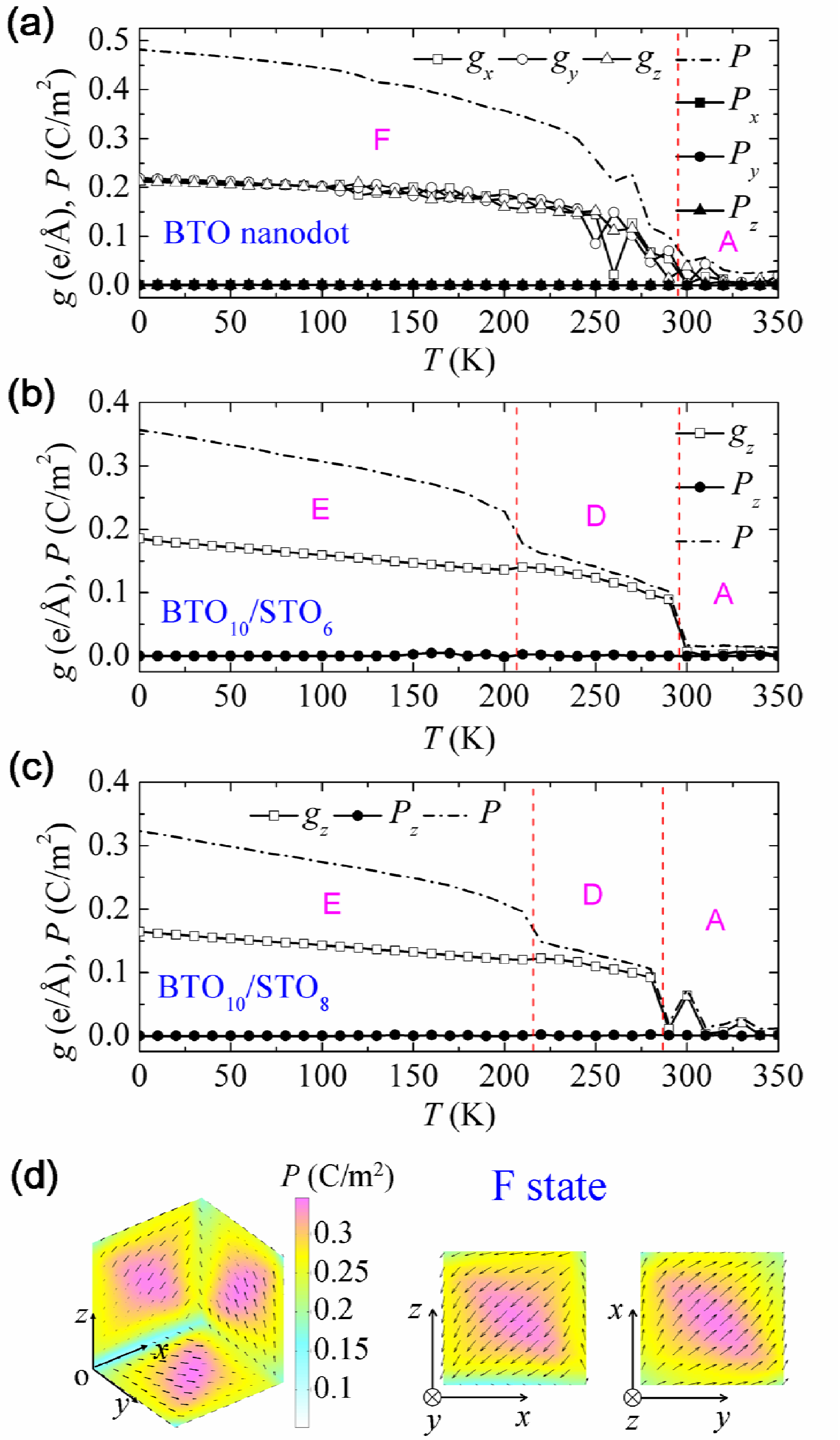

(a)
BTO nanodot
F
A
g_x g_y g_z P P_x P_y P_z
g (e/Å), P (C/m^2)
T (K)
(b)
BTO$_{10}$/STO$_6$
E
D
A
g_z P_z P
g (e/Å), P (C/m^2)
T (K)
(c)
BTO$_{10}$/STO$_8$
E
D
A
g_z P_z P
g (e/Å), P (C/m^2)
T (K)
(d)
P (C/m^2)
F state

Figure S2

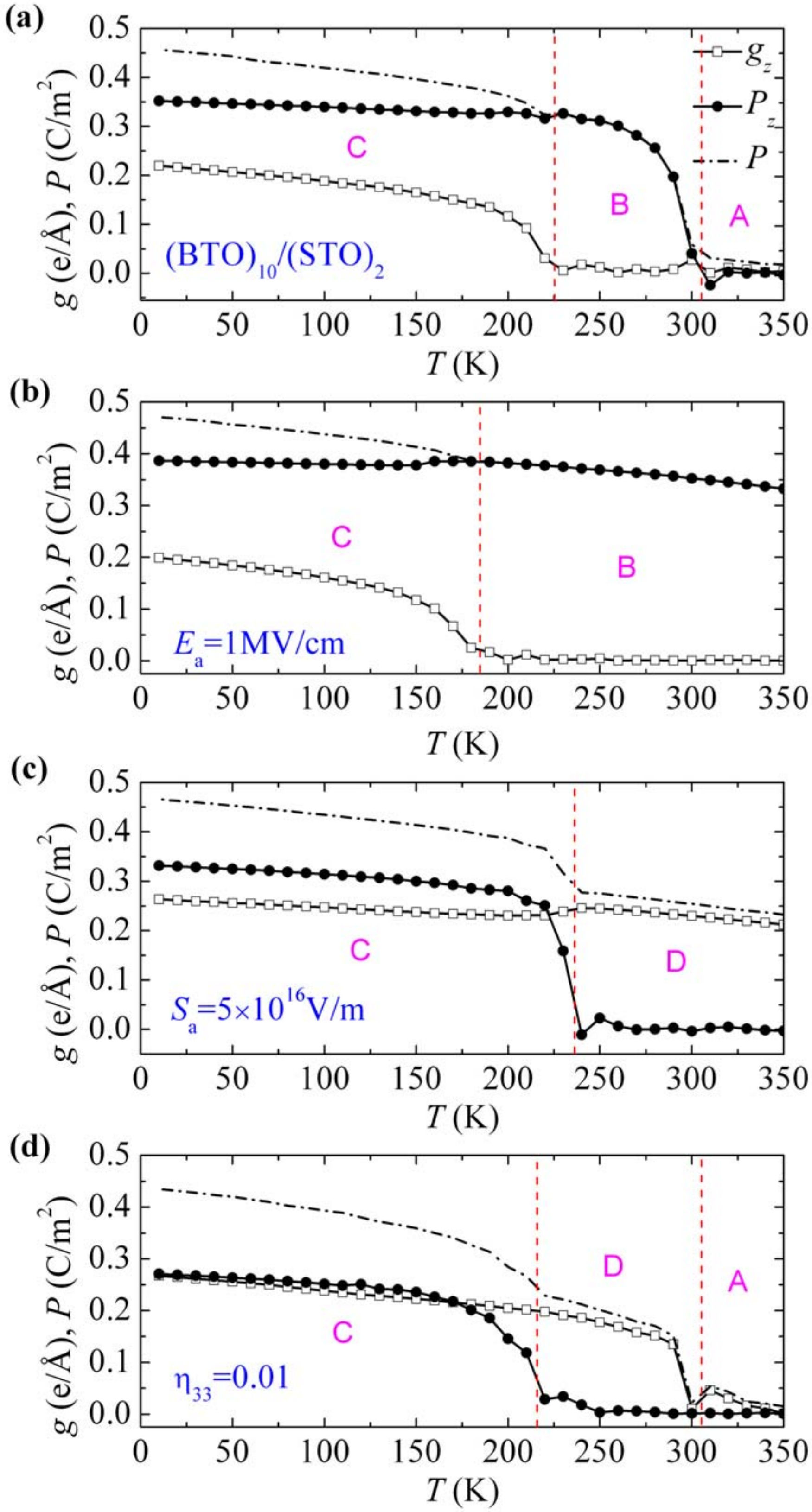
(a)
(BTO)10/(STO)2
C
B
A
gz
Pz
P
g (e/Å), P (C/m2)
T (K)
(b)
Ea=1MV/cm
C
B
(c)
Sa=5×10^16 V/m
C
D
(d)
η33=0.01
C
D
A

Figure S3

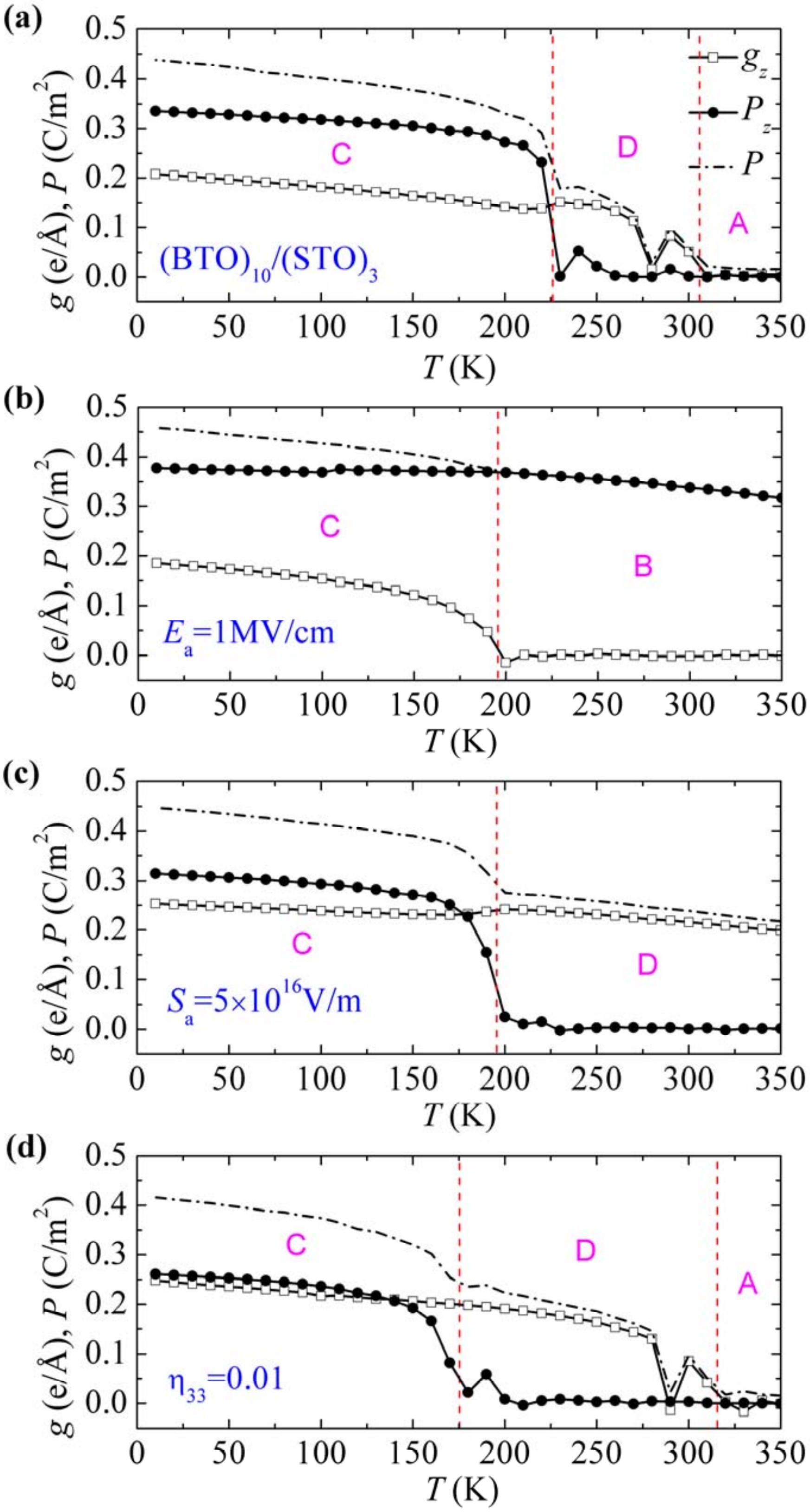
(a)
(b)
(c)
(d)
g (e/Å), P (C/m²)
T (K)
g_z
P_z
P
C
D
A
B
(BTO)10/(STO)3
Ea=1MV/cm
Sa=5×10^16V/m
η33=0.01

Figure S4

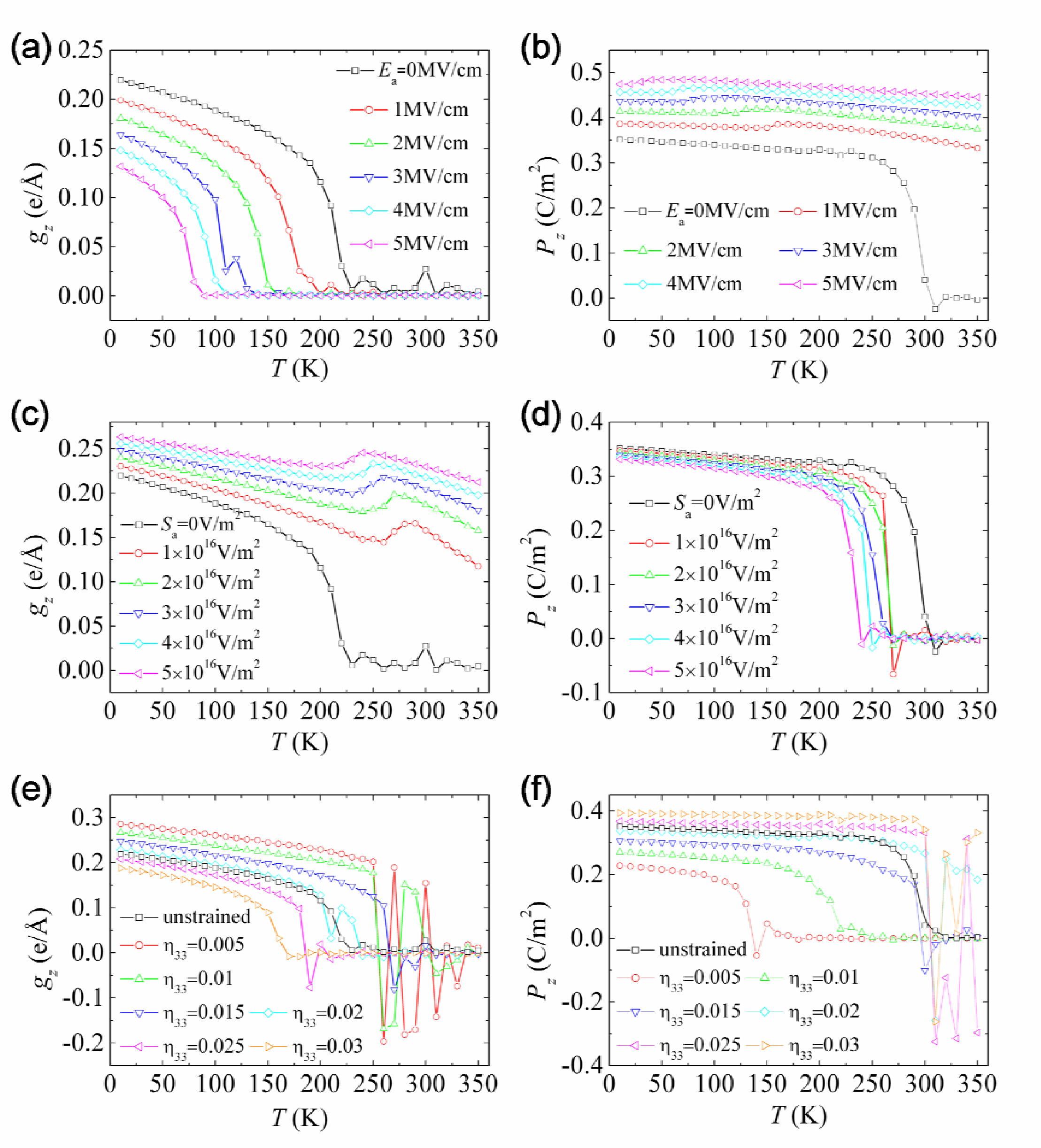

(a)
g_z (e/Å)
T (K)
E_a=0MV/cm
1MV/cm
2MV/cm
3MV/cm
4MV/cm
5MV/cm
(b)
P_z (C/m^2)
T (K)
E_a=0MV/cm
1MV/cm
2MV/cm
3MV/cm
4MV/cm
5MV/cm
(c)
g_z (e/Å)
T (K)
S_a=0V/m^2
1×10^{16}V/m^2
2×10^{16}V/m^2
3×10^{16}V/m^2
4×10^{16}V/m^2
5×10^{16}V/m^2
(d)
P_z (C/m^2)
T (K)
S_a=0V/m^2
1×10^{16}V/m^2
2×10^{16}V/m^2
3×10^{16}V/m^2
4×10^{16}V/m^2
5×10^{16}V/m^2
(e)
g_z (e/Å)
T (K)
unstrained
η_{33}=0.005
η_{33}=0.01
η_{33}=0.015
η_{33}=0.02
η_{33}=0.025
η_{33}=0.03
(f)
P_z (C/m^2)
T (K)
unstrained
η_{33}=0.005
η_{33}=0.01
η_{33}=0.015
η_{33}=0.02
η_{33}=0.025
η_{33}=0.03

Figure S5

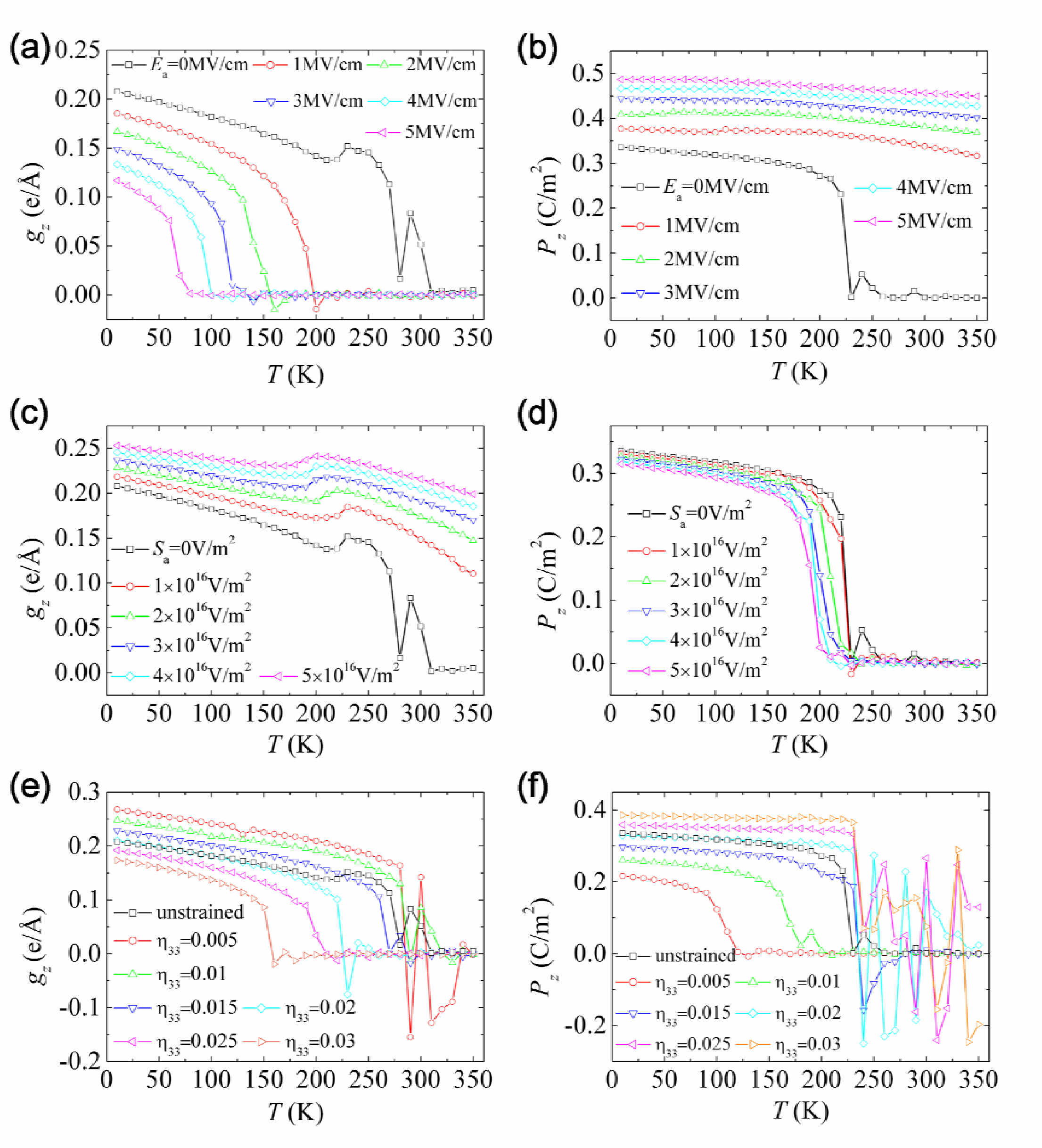

Figure S6

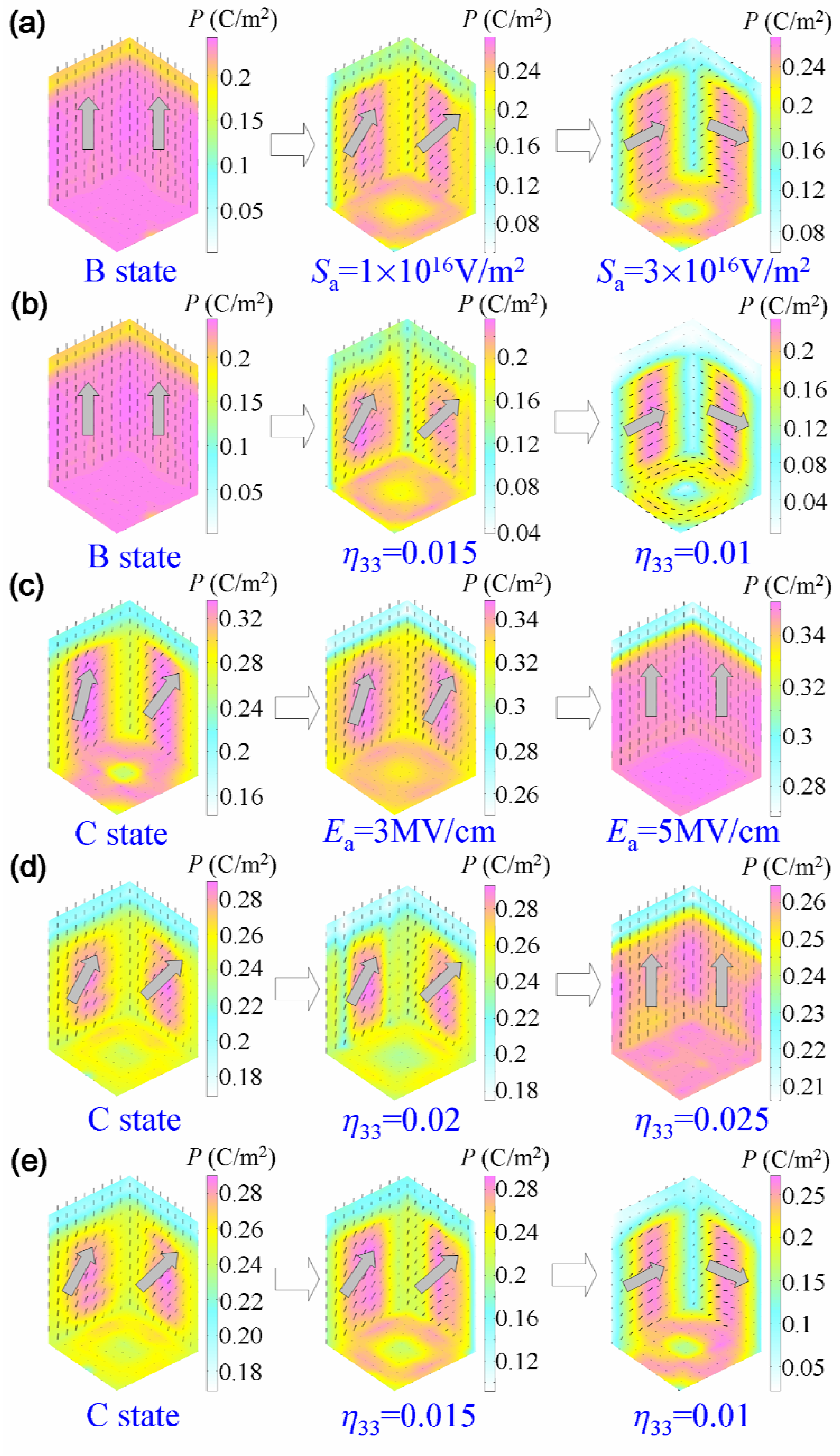
(a)
P (C/m²)
0.2
0.15
0.1
0.05
B state
P (C/m²)
0.24
0.2
0.16
0.12
0.08
S_a=1×10¹⁶V/m²
P (C/m²)
0.24
0.2
0.16
0.12
0.08
S_a=3×10¹⁶V/m²
(b)
P (C/m²)
0.2
0.15
0.1
0.05
B state
P (C/m²)
0.2
0.16
0.12
0.08
0.04
η_{33}=0.015
P (C/m²)
0.2
0.16
0.12
0.08
0.04
η_{33}=0.01
(c)
P (C/m²)
0.32
0.28
0.24
0.2
0.16
C state
P (C/m²)
0.34
0.32
0.3
0.28
0.26
E_a=3MV/cm
P (C/m²)
0.34
0.32
0.3
0.28
E_a=5MV/cm
(d)
P (C/m²)
0.28
0.26
0.24
0.22
0.2
0.18
C state
P (C/m²)
0.28
0.26
0.24
0.22
0.2
0.18
η_{33}=0.02
P (C/m²)
0.26
0.25
0.24
0.23
0.22
0.21
η_{33}=0.025
(e)
P (C/m²)
0.28
0.26
0.24
0.22
0.20
0.18
C state
P (C/m²)
0.28
0.24
0.2
0.16
0.12
η_{33}=0.015
P (C/m²)
0.25
0.2
0.15
0.1
0.05
η_{33}=0.01

Figure S7

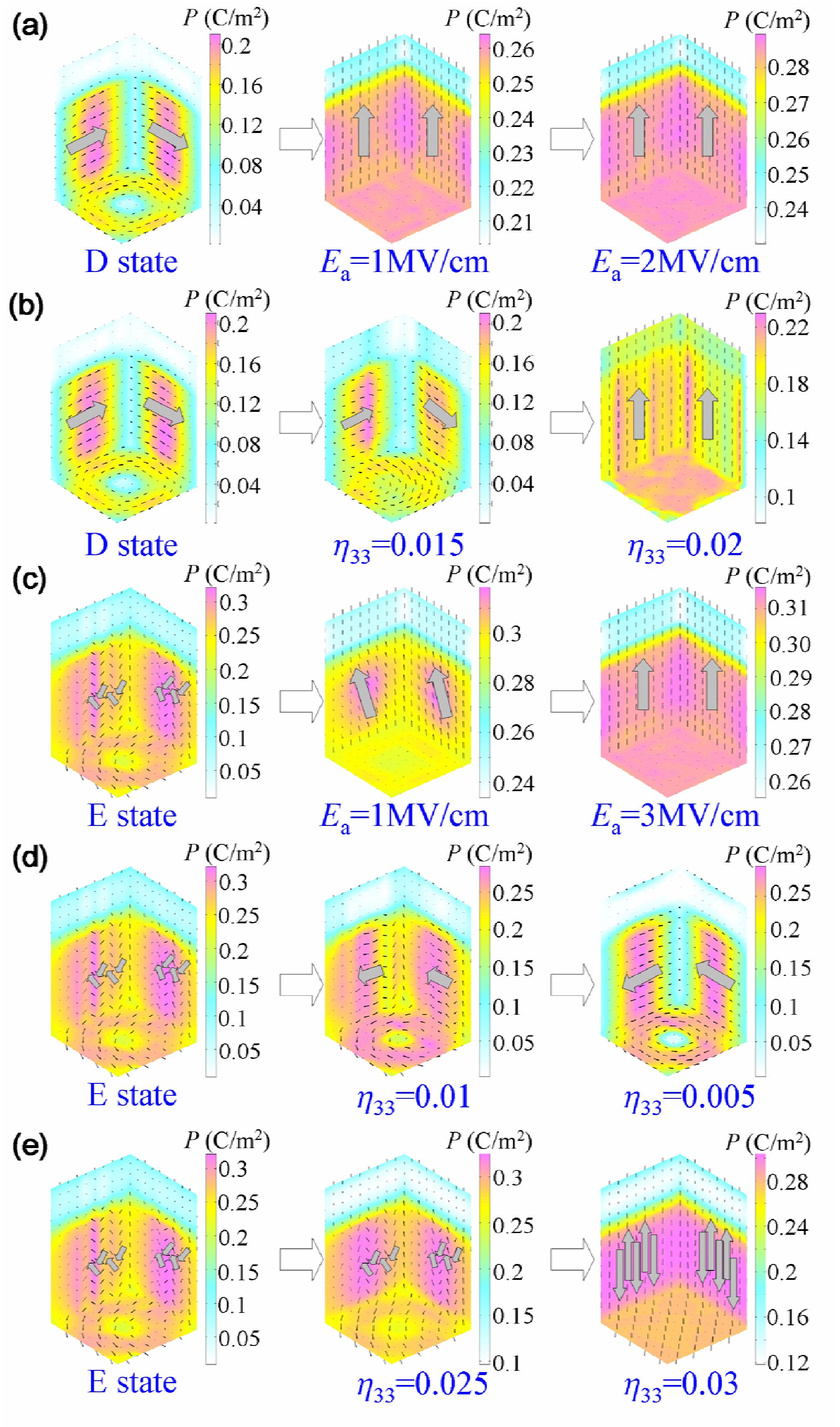

(a)
P (C/m2)
0.2
0.16
0.12
0.08
0.04
D state
0.26
0.25
0.24
0.23
0.22
0.21
Ea=1MV/cm
0.28
0.27
0.26
0.25
0.24
Ea=2MV/cm
(b)
0.2
0.16
0.12
0.08
0.04
D state
0.2
0.16
0.12
0.08
0.04
η33=0.015
0.22
0.18
0.14
0.1
η33=0.02
(c)
0.3
0.25
0.2
0.15
0.1
0.05
E state
0.3
0.28
0.26
0.24
Ea=1MV/cm
0.31
0.30
0.29
0.28
0.27
0.26
Ea=3MV/cm
(d)
0.3
0.25
0.2
0.15
0.1
0.05
E state
0.25
0.2
0.15
0.1
0.05
η33=0.01
0.25
0.2
0.15
0.1
0.05
η33=0.005
(e)
0.3
0.25
0.2
0.15
0.1
0.05
E state
0.3
0.25
0.2
0.15
0.1
η33=0.025
0.28
0.24
0.2
0.16
0.12
η33=0.03

Figure S8

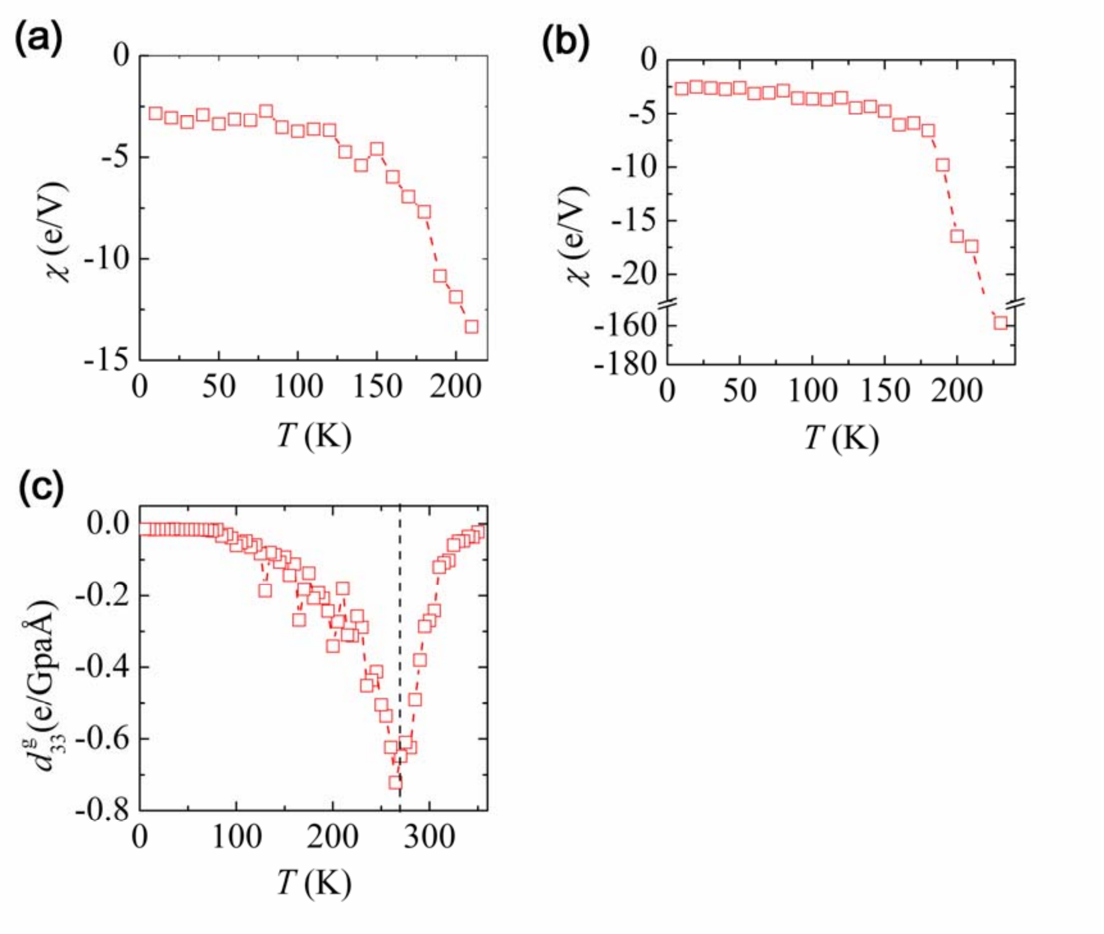
(a)
χ (e/V)
T (K)
0
-5
-10
-15
0
50
100
150
200
(b)
χ (e/V)
T (K)
0
-5
-10
-15
-20
-160
-180
0
50
100
150
200
(c)
d^g_{33}(e/GpaÅ)
T (K)
0.0
-0.2
-0.4
-0.6
-0.8
0
100
200
300

Figure S9

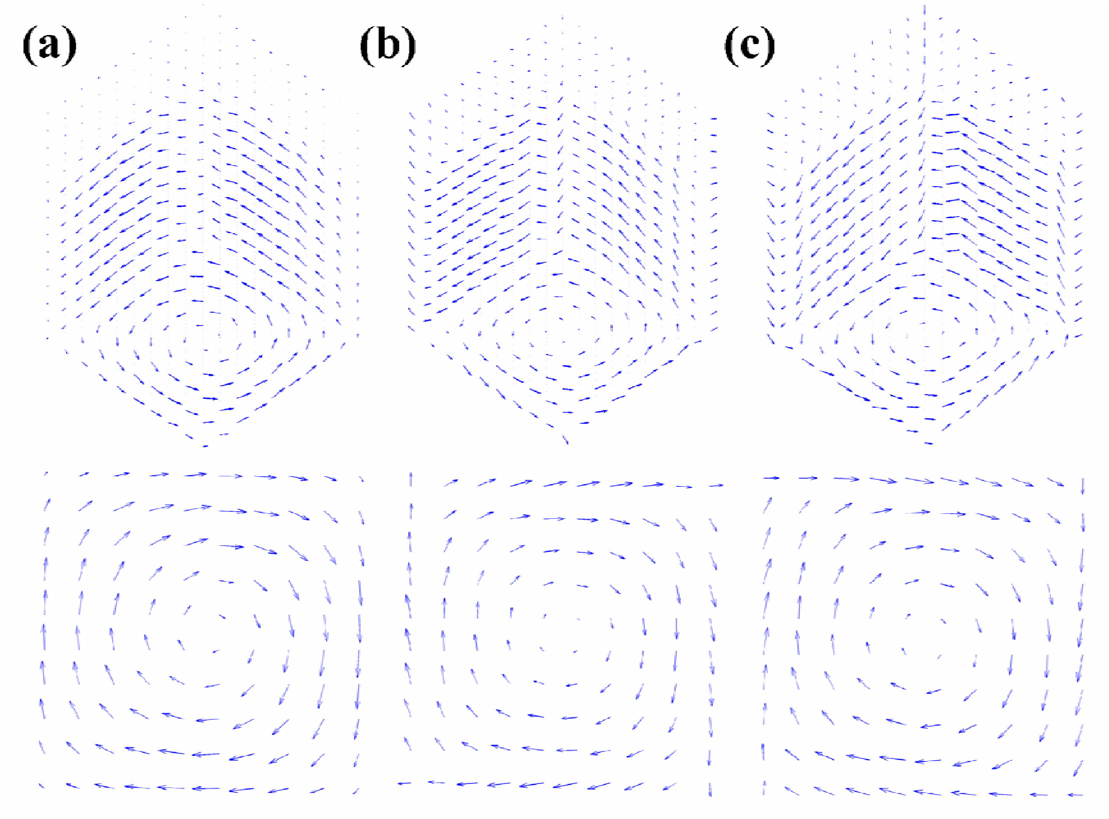

Figure S10

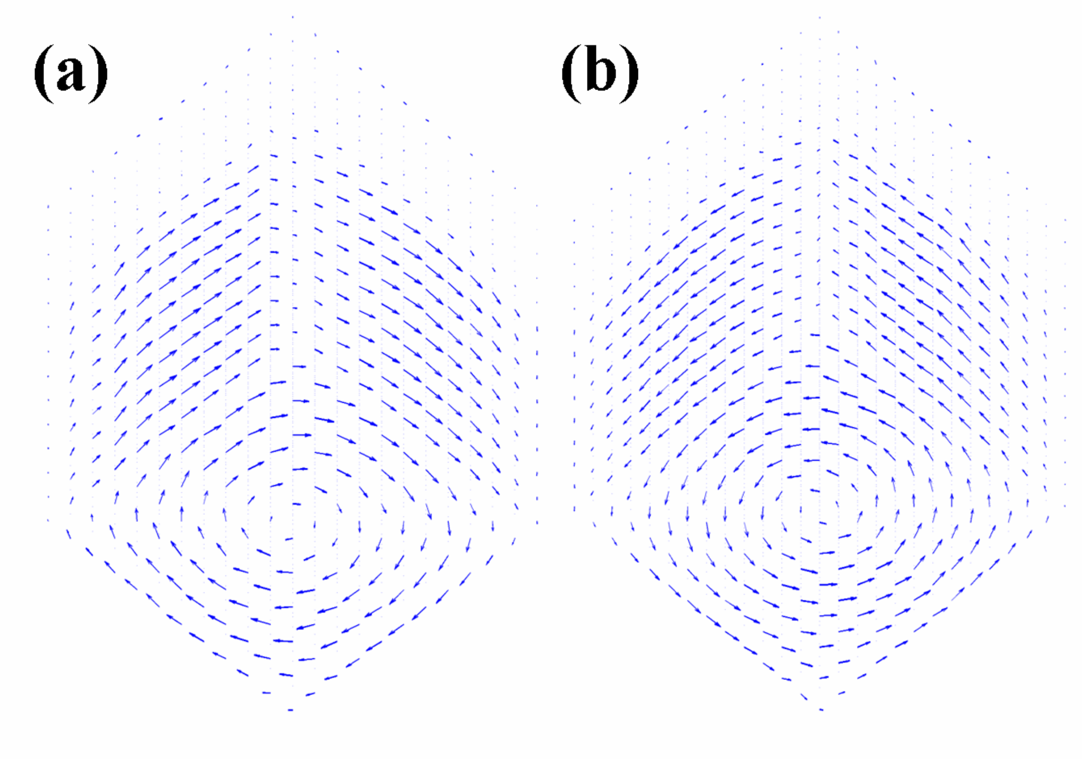

Supplement: Supplementary Information [file srep11165-s1.pdf]
